# Association Between Proinflammatory Cytokines IL-6 and TNF-Alpha, Psychological Stress and Chronic Spontaneous Urticaria Severity

**DOI:** 10.3390/ijms262110384

**Published:** 2025-10-25

**Authors:** Liborija Lugović-Mihić, Maja Štrajtenberger, Matea Kuna, Blaženka Ladika-Davidović, Ema Barac, Maja Vilibić

**Affiliations:** 1 School of Dental Medicine, University of Zagreb, 10000 Zagreb, Croatia; matea.kuna43@gmail.com; 2 Department of Dermatovenereology, University Hospital Center “Sestre Milosrdnice”, Vinogradska cesta 29, 10000 Zagreb, Croatia; 3 Department of Pulmology, Special Hospital for Pulmonary Diseases, 10000 Zagreb, Croatia; dr.strajs@gmail.com; 4 Department of Oncology and Nuclear Medicine, University Hospital Center “Sestre Milosrdnice”, Vinogradska 29, 10000 Zagreb, Croatia; blazenka.ladika.davidovic@kbcsm.hr; 5 Family Physician Office, 10000 Zagreb, Croatia; ema.barac@gmail.com; 6 Department of Psychiatry, University Hospital Center “Sestre Milosrdnice”, 10000 Zagreb, Croatia; maja.vilibic@gmail.com; 7 School of Medicine, Catholic University of Croatia, 10000 Zagreb, Croatia

**Keywords:** chronic urticaria, IL-6, TNF-α, cortisol, molecules, stress, chronic spontaneous urticaria, disease severity, interleukins, cytokines, diagnostics, disease severity, Urticaria Activity Score, inflammation, treatment, psychological factors, perceived stress, quality of life, disease management

## Abstract

Chronic urticaria (CU), defined as the appearance of wheals/angioedema lasting ≥6 weeks, is often associated with psychological factors like stress. Stress-induced reactions involve the psychological–neuroendocrine–immunological network, which influences disease course/outcome and patient quality of life (QoL). With 46 participants (23 with CU and 23 healthy controls/HCs), this research examined the relationship between values of serum proinflammatory cytokines (IL-6 and TNF-α), stress indicators (cortisol levels, perceived stress level), and clinical chronic spontaneous urticaria (CSU) features (CSU severity/UAS, patient QoL). For CSU patients, significantly higher levels of IL-6 (*p* = 0.002) and TNF-α (*p* = 0.001) were recorded, as well as higher cortisol levels (*p* = 0.015) and a lower perception of stress/PSS (*p* < 0.001) than for HCs. CSU severity linearly and positively correlated with serum cortisol level (r = 0.463; *p* = 0.463) and impaired QoL (r = 0.715; *p* < 0.001). Additionally, impaired QoL correlated positively with perceived stress (r = 0.523; *p* = 0.010) and negatively with age (r = −0.529; *p* = 0.009). Also, IL-6 levels negatively correlated with perceived stress (r = −0.402; *p* = 0.006) linearly and moderately. The significant negative correlation between psychological stress and CU indicates that a comprehensive approach to treatment is necessary.

## 1. Introduction

Chronic urticaria (CU), common inflammatory skin disease, significantly affects patients due to its unpredictability, associated problems in daily life, persistence, and occasional therapeutic inefficacy, which can all pose a therapeutic challenge [1,2]. CU’s recognizable characteristics are wheals/angioedema, which last six weeks or more. Chronic spontaneous urticaria (CSU) is the most common form of CU, and its wheals are often accompanied by intense pruritus [3,4]. Patients experience sleep disturbances and have a reduced quality of life and a higher propensity for psychiatric comorbidity and/or psychological burden, such as depression and anxiety [5,6,7,8,9]. Although not life-threatening, symptoms of CSU significantly affect various aspects of everyday life [1,2].

In the pathogenesis of CU, skin mast cells and basophils are key inflammatory cells that, after being stimulated by IgE, are activated and release proinflammatory mediators, including histamine, leukotriene C4, platelet-activating factor (PAF), IL-13, IL-25, CXCL8/IL-8, and others [10,11,12,13,14,15,16]. Thus, mast cells secrete many preformed mediators (such as tryptase, heparin, chymase, carboxypeptidase A3, cathepsin G, renin, etc.) and prostaglandin D2. Binding of histamine to its receptor H4R triggers mast cell activation, which involves stimulation of intracellular calcium mobilization and ultimately leads to the expression of proinflammatory mediators like tumor necrosis factor-alpha (TNF-α), IL-4, IL-5, IL-6, IL-8, tumor growth factor β1 (TGF-β1), macrophage inflammatory protein 1α (MIP-1α), activation-regulated, normally expressed and secreted by T-cells (RANTES) and monocyte chemoattractant protein 1 (MCP-1). Thus, activated mast cells release mediators associated with inflammation (histamine, TNF-α, MMP-9, IL-4, IL-6 and others), important for T cell-mediated inflammation and extravasation and recruitment of leukocytes to the area of skin inflammation. Consequently, significantly higher serum levels of histamine, leukotriene C4, TNF-α, IL-4, IL-5, IL-6 and TGF-β have been confirmed in CU subjects than controls (mast cells produce significant amounts of the cytokines IL-6 and IL-5) [10,11,12,13,14,15,16]. However, it should be mentioned that many other factors and pathogenetic processes participate in the complex pathogenesis of chronic urticaria [17,18,19,20,21,22].

Research has shown that patients with CSU often exhibit elevated inflammatory markers and mediators. According to the recommendations for CSU, in diagnostic procedures it is necessary to evaluate erythrocyte sedimentation rate (ESR), differential blood count (DBC), and/or C-reactive protein (CRP), IgG anti-TPO and total IgE values [1,22]. In addition, increased serum levels of proinflammatory cytokines IL-6 and TNF-α have been observed in CU patients. These proinflammatory cytokines are considered potential biomarkers of CSU, as their plasma levels are elevated in patients with more active disease and significantly lower during spontaneous remission. IL-6, as a proinflammatory cytokine, produced predominantly by Th2 cells, participates in immune pathways and promotes Th2 differentiation, indirectly inhibiting Th1 differentiation. It is important to mention that, while Th1 cells produce cytokines IL-2, TNF-α, interferon-gamma (IFN-γ), etc., Th2 cells produce many other cytokines (IL-4, IL-5, IL-6, IL-10, IL-13). Thus, Th1 cells primarily support cell-mediated immunity (e.g., elimination of infected cells), while Th2 cells promote humoral immunity (activating B cells, stimulating production of antimicrobial antibodies, etc.). This involvement of immunological factors implies that CSU is an immune-mediated chronic inflammatory condition arising from immune activation following exposure to various triggering factors [23,24,25,26].

Among the triggers of, and influences on, CU occurrence and exacerbations, the negative impact of psychological stress is frequently reported, either when observed in CU patients by healthcare professionals or when reported by patients themselves [22,27]. The stress response involves physiological mechanisms primarily governed by two neuroendocrine systems: the sympatho-adrenal–medullary (SAM) system and the hypothalamic–pituitary–adrenal (HPA) axis. In response to perceived stress, the hypothalamus releases corticotropin-releasing hormone (CRH), stimulating the anterior pituitary to secrete adrenocorticotropic hormone (ACTH), subsequently leading to the release of cortisol, a biomarker for stress level, from the adrenal cortex [27,28,29,30,31,32]. According to literature data, psychological stress influences skin condition, involving a network of various factors and a link between skin hormones, immune factors and epidermal structures (keratinocytes) and dermal structures (fibroblasts, mast cells) and their receptors for hormones and immune factors. In these processes, T cells have a very important role, predominantly Th1 and Th2 cells. Specifically, Th2-derived cytokines like IL-6 and Th1-derived TNF-α are important factors for cross-talk between skin structures and immune systems during the skin stress response (including cortisol) [31]. Also, during psychological stress, released stress hormones and neuropeptides (e.g., CRH and substance P) bind to mast cell receptors and stimulate them to release their mediators, thus leading/contributing to many inflammatory and allergic conditions. Thus, stress-induced mast cell activation and the release of their inflammatory mediators may support neuroinflammation, which has a negative influence on skin and brain functions. Consequently, psychological stress may deteriorate allergic conditions through this process.

The aim of this research was to investigate the relationship and correlation between clinical features of CSU (disease severity and quality of life) and levels of serum proinflammatory cytokines (IL-6 and TNF-α), as well as stress levels (serum cortisol levels and perceived psychological stress), by comparing CSU patients and healthy controls (HCs). Additionally, the study aimed to determine the intercorrelation of the measured parameters and their relationship with CSU severity and to identify differences in the measured factors between CSU patients and HCs.

## 2. Results

Analysis of participants by age and sex revealed no significant differences between the patient and control groups. Furthermore, no differences in the examined parameters were found between male and female participants. The levels of the examined serum cytokines and cortisol and perceived stress were not associated with age in the total sample.

**Figure 1:**
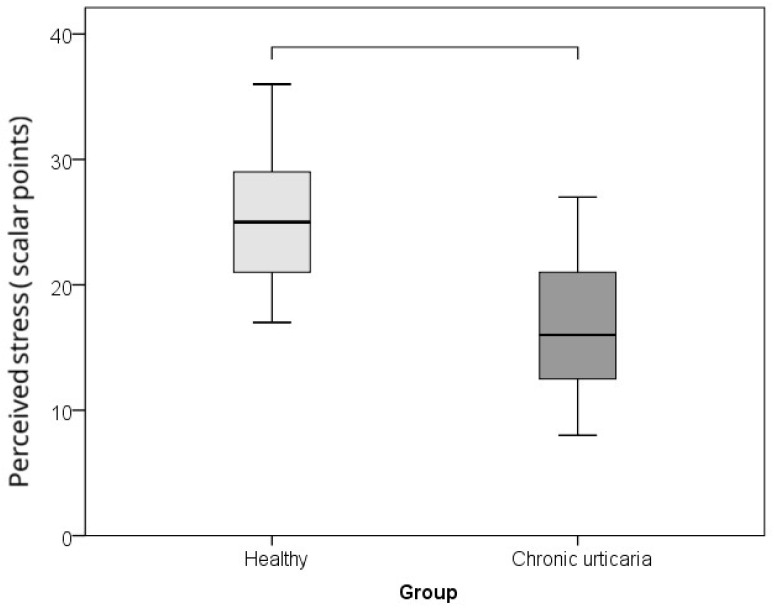
Comparison between healthy controls and CSU patients for perceived stress levels (horizontal lines denote significant differences between groups).

**Figure 2:**
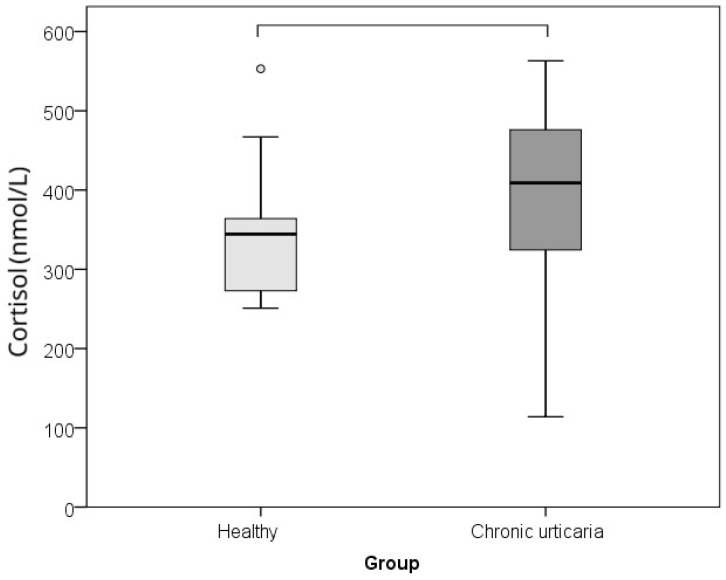
Comparison between healthy controls and CSU patients for serum cortisol values (horizontal lines denote significant differences between groups; circles represent outliers).

**Figure 3:**
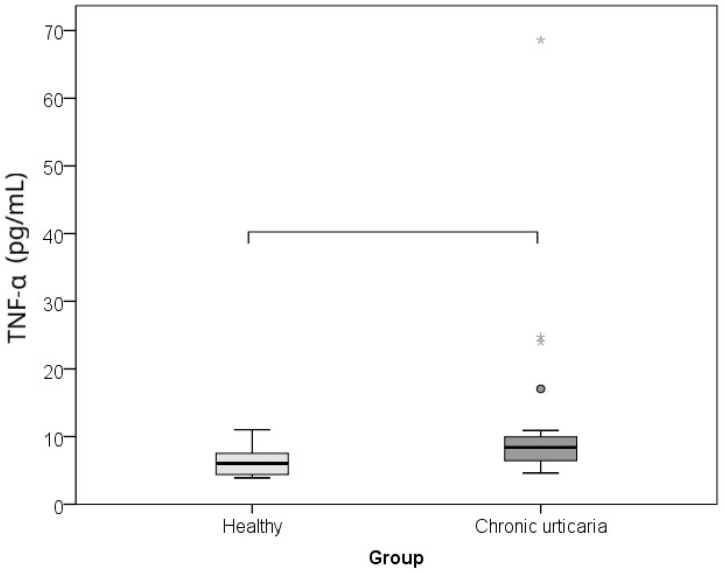
Comparison between healthy controls and CSU patients for serum TNF-α levels (horizontal lines denote significant differences between groups; circles represent outliers and stars extremes).

**Figure 4:**
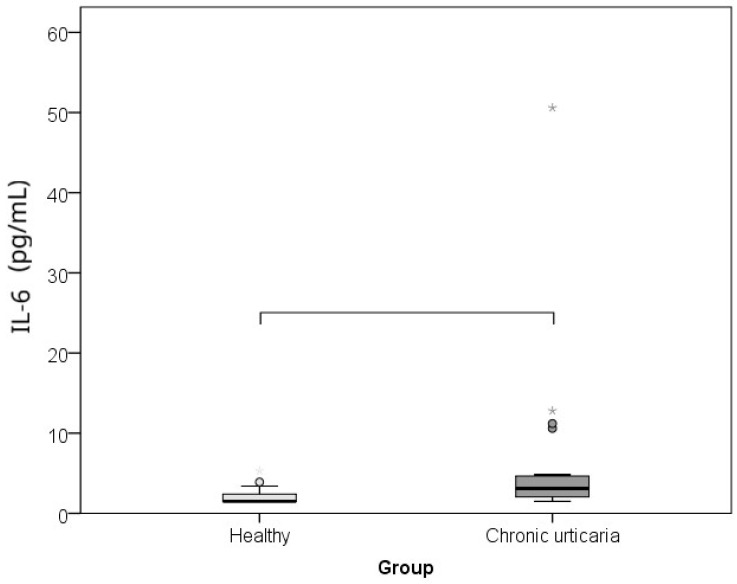
Comparison between healthy controls and CSU patients for serum IL-6 values (horizontal lines denote significant differences between groups; circles represent outliers and stars extremes).

The comparison between CU patients and healthy controls of serum levels of IL-6, TNF-α, and morning cortisol, as well as perceived psychological stress (PSS), showed that subjects with CU had significantly higher levels of IL-6 (*p* = 0.002; r = −0.460), TNF-α (*p* = 0.001; r = −0.485), cortisol (*p* = 0.015; r = −0.360), and lower perceived stress/PSS (*p* < 0.001; r = −0.608) than healthy individuals (Table 1; Figure 1, Figure 2, Figure 3 and Figure 4). The effect was large for perceived stress and moderate for cortisol, IL-6 and TNF-α.

The analysis of the correlation between serum levels of IL-6, TNF-α, and morning cortisol levels with disease severity and quality of life in patients with CSU revealed the following: The severity of CSU showed a positive linear correlation with serum cortisol levels (r = 0.463; *p* = 0.046) and with impaired quality of life (r = 0.715; *p* < 0.001). Additionally, impaired quality of life was positively correlated with perceived stress (r = 0.523; *p* = 0.010) and negatively correlated with age (r = –0.529; *p* = 0.009) (Table 2).

When looking at correlations for both healthy persons and CSU patients, IL-6 correlated with perceived stress (r = −0.402; *p* = 0.006) linearly, negatively and moderately. As stress increased, IL-6 decreased. There were no associations between other parameters. In healthy individuals, no significant correlations were detected between serum markers, stress, and age.

## 3. Discussion

Psychological stress is often reported as a trigger for, or contributing factor to, chronic dermatoses such as CU, yet research on this topic remains limited. One major challenge lies in accurate objectivization of stress, which can vary significantly between individuals. For this, psychometric questionnaires are valuable tools for assessing psychological stress, primarily by evaluating changes in behavior and cognitive functions. Additionally, serum cortisol, the most used biomarker of stress, is also frequently measured. In recent years, scientific interest has increasingly focused on the psychoneuroimmunological (PNI) approach, which explores the impact of stress on disease onset and progression [31,33,34]. However, despite considerable theoretical insight, clinical studies with dermatological patients that simultaneously examine psychological, neuroendocrine, and immune parameters—and their interrelations—remain scarce. This gap in the literature was a key motivating factor for our study. Our findings show that CSU patients exhibited significantly higher serum cortisol levels, but lower perceived stress (PSS) scores compared to HCs. This suggests that, although CSU patients exhibit a significant biologically measurable level of stress, their subjective perception of stress may be reduced. This could be, at least partially, explained by the mobilization of positive coping and adjustment mechanisms due to the long duration of CSU. Notably, CSU severity in our sample was positively correlated with cortisol levels and associated with poorer quality of life, implying a meaningful link between disease severity, stress level, and quality of life impairment. This aligns with existing evidence indicating a bidirectional relationship between chronic illnesses like CU and psychological stress: chronic skin conditions may induce stress, while chronic stress can exacerbate or trigger disease activity [30,34,35,36,37].

The stress response involves the adrenal and sympathetic components of the neuroendocrine system, both of which exert significant influence on immune function [31]. Lymphoid tissues receive signals via sympathetic and parasympathetic neurons, thereby modifying immune cell behavior. The immune system comprises both innate and adaptive components, including cellular and humoral immunity, coordinated by cytokine mediators that facilitate intercellular communication. Under stress, ACTH stimulates adrenal cortisol production. Unlike the immediate effects of sympathetic activation and adrenaline release, prolonged cortisol exposure may impair immune functioning depending on stress intensity and duration [31,35]. CU is known to be associated with an increase in proinflammatory cytokine production, and these cytokines contribute to local skin inflammation and recruit immune cells involved in lesion resolution. Some also activate the sympathetic nervous system, promoting catecholamine release. Among these, IL-1, IL-6, and TNF-α are particularly important for HPA axis activation and for initiating and sustaining skin inflammatory responses [38]. The type and duration of psychological stress are also crucial. Acute stress, through HPA axis activation and elevated cortisol, may suppress proinflammatory cytokines (IL-1, IL-6, TNF-α), whereas chronic stress can induce cortisol resistance, paradoxically resulting in elevated cytokine levels [39]. Psychological stress (acute or chronic) influences skin T cell populations and affects cutaneous Th1/Th2 balance. Thus, chronic stress, such as the stress from chronic dermatoses like CSU, increases the values of stress-related hormones like cortisol, which may suppress Th1-mediated immune reactions and promote Th2 responses [32]. Acute stress, on the one hand, often leads to a predominant Th1 response (cellular immunity), while chronic stress, on the other, often leads to a Th2 response (humoral immunity). This Th1/Th2 shift/imbalance and Th2 promotion may deteriorate allergic and autoimmune conditions such as CSU. In our study, the duration of CSU was not associated with cytokine levels or any other measured variable.

While some previous studies have shown reduced cortisol levels in CU patients, an inverse correlation has also been observed between cortisol and psychological stress intensity [36]. In our study, CSU patients demonstrated higher cortisol levels and lower perceived stress, although these two variables did not correlate. Previous studies also reported that IL-18 and CRP (regulated by cortisol) positively correlated with CSU severity (measured by the UAS), whereas cortisol showed a negative correlation with CSU severity, which is consistent with our findings [37]. These results suggest that chronic stress in CU patients may reduce cortisol levels and elevate inflammatory mediators [37]. Additionally, in CSU patients, impaired quality of life was positively correlated with perceived stress and negatively with age. This implies that both stress perception and younger age negatively impact quality of life. Younger adults could exhibit less adaptive stress-coping capacities and also experience greater disruption in work, family, or educational responsibilities due to disease burden. Moreover, IL-6 was significantly associated with perceived stress, showing a moderate negative linear correlation—as perceived stress increased, IL-6 levels decreased. This finding indicates a potential link between proinflammatory cytokines and stress in CSU manifestations.

Consistent with other studies, we confirmed that IL-6 and TNF-α serum levels were significantly greater for those with CSU than for healthy controls (*p* = 0.002, *p* = 0.001). In a study by Grzanka et al., those with moderate or severe CSU had higher TNF-α levels, but no significant difference was seen between those with mild CSU and healthy individuals [40]. Genetic predispositions have also been suggested [20,21,41]. For instance, Tavakol et al. found that polymorphisms in the TNF-α and IL-6 genes may influence CU susceptibility. These cytokines play roles in the initiation, course and progression of inflammatory and autoimmune diseases such as CU [41]. Thus, a feedback loop of sustained inflammation is created when TNF-α and IL-6 activate immune cells (these cytokines, which are secreted by macrophages and activated inflammatory cells, also control apoptosis and cellular differentiation) [41]. While Habal et al. found no significant differences in IL-6 or TNF-α levels between patients with CU/angioedema and controls, other studies have demonstrated clinical efficacy of TNF-α inhibitors in CU patients [42,43,44]. In our cohort, IL-6 levels were significantly elevated and negatively correlated with perceived stress, reinforcing the complexity of these interactions. CU patients have also been shown to exhibit elevated psychological stress levels [45], which our findings support, as indicators/markers of stress between those with CSU and healthy controls were significantly different. When interpreting these results, it is important to recognize and highlight the bidirectional relationship between chronic diseases and stress, where stress may be both a cause and a consequence [30,34].

The negative correlation between psychological stress and CU indicates a comprehensive approach to treatment is necessary. Regarding therapeutic implications, for instance, antidepressants have demonstrated anti-inflammatory effects and reduction in skin inflammation and urticarial symptoms in chronic skin disorders, suggesting their potential use beyond psychiatric comorbidities [46]. In addition to the key medications for CSU (antihistamines, omalizumab, cyclosporine), systemic corticosteroids are also useful (for a short time) for exacerbation, which may influence their cortisol levels and patients’ stressful states and their HPA axis [47]. Concerning patient management, a potential treatment option for the prevention/resolution of stress-associated inflammation could be targeting mast cells and inhibiting their stimulation by neuropeptides (e.g., CRH). In addition, lifestyle changes that reduce stress, including exercise, sufficient sleep, rest, appropriate nutrition, could good improve a patient’s skin condition. Furthermore, when considering the stress-dermatoses link, it should be taken into account their cyclic connection and bidirectional relationship, where stress exacerbates dermatoses, and the dermatoses, in turn, burden the patient both physically and psychologically aspects, further contributing to stress, as well as possible depression and anxiety. Thus, the involvement of a psychologist or psychiatrist can sometimes be beneficial for those prone to stress-associated dermatoses [48]. The cycle described above underlines the need for a multidisciplinary and interdisciplinary approach to patients and treatment [49,50,51]. However, to improve treatment, more studies are necessary for better insight into the pathomechanisms of stress-mediated mast cell activation, a key player in CSU.

To our knowledge, this is the first study that has simultaneously compared stress levels and proinflammatory markers in the same CSU patient group with healthy controls, while also considering disease severity. Its predominant limitation is the small number of patients. Still, this small sample detected significant differences between groups and correlations between variables. Also, in the analysis of CSU patients’ stress, potential treatment-related stress and previous corticosteroid treatments were not taken into account. Likewise, the bidirectional relationship between stress and CU could be analyzed in further studies. Also, we considered only three serum immune factors, but there are CU biomarkers (e.g., ESR and CRP) which could be correlated with the stress levels, other proinflammatory markers and CSU severity. In addition, it could be useful to include more data on stress and CSU patients specifities, e.g., the relationship between stress levels (and proinflammatory markers) and age and gender (in our study, females predominated, which is in accordance with epidemiological data that CSU is more common in females). However, considering that are very few studies which have analyzed stress levels and proinflammatory mediators, this research may be a basis for further studies that include a larger number of patients.

Our findings support the existence of significant differences in immunological, neuroendocrine, and psychological parameters between CSU patients and healthy individuals. They also provide insight into how these factors relate to disease severity and patient quality of life. Understanding these correlations contributes to a better grasp of CSU pathogenesis and highlights opportunities for future research and a more effective, multidisciplinary therapeutic approach, ultimately improving outcomes and quality of life in affected patients. The identification of serological biomarkers for urticaria activity, and the clarification of their relationship with psychological states such as stress, remain priority areas for further research, as mentioned in current trends and perspectives for CU [1].

## 4. Materials and Methods

### 4.1. Methodology

We performed a cross-sectional case–control study at the University Hospital Center “Sestre Milosrdnice”, Department of Dermatology and Venereology. The study included patients with CSU who were triaged during dermatological evaluations at the Allergy and Clinical Immunology Outpatient Clinic. All participants were examined by a board-certified dermatologist. The control group consisted of healthy individuals, matched to the CSU patients by age and sex, who met the inclusion criteria. Each participant was informed about the study protocol, and written informed consent was obtained prior to participation. The Ethics Committee for the University Hospital Center “Sestre Milosrdnice” in Zagreb, Croatia approved the study on January 13, 2022 (Protocol number: 251-29-11-22-01-9).

### 4.2. Participants

Participants were recruited from a pool of CSU follow-up patients who met all the inclusion criteria (18 plus years of age, CSU diagnosis based on clinical guidelines for dermatological examination, and a patient history involving wheals and/or angioedema lasting more than 6 weeks) [1]. Patients with isolated angioedema or isolated chronic inducible urticaria were excluded.

Exclusion criteria for all participants included use of psychoactive medications, corticosteroids, or immunosuppressants within one month prior to enrollment; vaccination within 28 days prior to study commencement; breastfeeding; use of oral contraceptives; being pregnant or currently breastfeeding; follicular phase of the menstrual cycle; having a systemic inflammatory/autoimmune disease; diseases of the oral mucosa; and a medical history including neoplasms and psychiatric disorders.

Inclusion criteria for healthy controls were an age of 18 years or over and the absence of skin, inflammatory, or autoimmune diseases.

In total, the study included 46 participants: 23 patients with diagnosed CSU and 23 healthy controls. The sample consisted of individuals aged 21 to 73 years (median age: 35; interquartile range: 28–48), with 30 out of 46 (65%) being female. For each patient with CSU, we also recorded disease duration (in months).

### 4.3. Questionnaires

Disease severity in CSU patients was evaluated using the Urticaria Activity Score (UAS7) and the Dermatology Life Quality Index (DLQI) [1].

The Urticaria Activity Score (UAS) asks respondents to assess both urticaria (hives) and pruritus (itching) severity during the previous 24 h. Each are scored from 0 to 3, 0 indicating an absence of symptoms, 1 being mild, 2 being moderate, and a score of 3 indicating severe severity. The UAS7 assesses severity on a 1 to 42-point scale over a period of 7 days (1–6 points: well-controlled CSU; 7–15: mild CSU; 16–27: medium severity of CSU; and 28–42 points: severe CSU [1,52].

The Dermatology Life Quality Index (DLQI): On this 10-question survey, respondents are asked to assess the impact of their disease over the previous week on certain areas of their life—symptoms and feelings, daily activities, free time, work and school, personal relationships, and treatment. Each question is scored from 0–3. A total score of 0 or 1 suggests the disease had no effect on the patient, while 2–5 points would suggest a small effect, 6–10 would be a moderate effect, 11–20 would be a large effect, and a score of 21–30 would indicate a very large impact [53].

### 4.4. Serum Biomarker Analysis

Blood sample collection occurred between 7:00 a.m. and 9:00 a.m. From these samples, proinflammatory cytokine (IL-6, TNF-α) levels and cortisol levels were analyzed.

Serum concentrations of IL-6 and TNF-α were measured using the chemiluminescence method on an Immulite 1000 analyzer. Analyses were performed at the Department of Oncology and Nuclear Medicine, University Hospital Center “Sestre Milosrdnice”, using the Immulite 1000 (Siemens Healthcare Diagnostics Inc., Flanders NJ, USA), and FACScalibur (flow cytometry; BD FACScalibur; BD Becton Dickinson, Franklin Lakes, NJ, USA) analyzers.

The reference values for serum cytokines IL-6 and TNF-α (IMMULITE^®^ 1000 and FACSCalibur™) were as follows: for IL-6: 0–7 pg/mL, while TNF-α: 0–8.1 pg/mL [50].

An electrochemiluminescent immunoassay (ECLIA) was used to measure serum cortisol levels. The reference values for measured cortisol levels (Roche, ECLIA) were 133–537 nmol/L.

### 4.5. Psychological Stress Analysis

Psychological stress was assessed both by measuring serum cortisol levels and by using the Perceived Stress Scale (PSS) questionnaire [54,55,56,57,58].

All participants, including the healthy controls, answered the PSS survey that asks respondents to recall the stress they experienced over the month preceding the survey. The questionnaire includes 10 questions scored on a Likert scale from 0–4. The total score falls into three groups of stress experience: low stress (0–13 points), moderate stress (14–26 points), and high stress (27–40 points).

### 4.6. Statistical Analysis

Since there were fewer than 30 respondents per group, we used non-parametric statistics, the Mann–Whitney test to analyze continuous data. To compare frequencies, we used the Fisher test, and to calculate effect size, r = Z/√N was used for Mann–Whitney and Cramer’s V was used for the Fisher test. The relationship between the continuous variables was checked by analyzing the scatter plot and Spearman’s correlation. Effect size and correlation strength were interpreted using Cohen’s criteria: r = 0.25–0.3 = small effect size /low correlation, 0.3–0.5 = moderate, 0.5–0.7 = large, and >0.7 = very large. Analyses were performed using the IBM SPSS 22 (IBM Corp., Armonk, NY, USA) commercial software.

## 5. Conclusions

In CSU patients, significantly elevated proinflammatory cytokine levels of IL-6 and TNF-α, higher cortisol levels, and lower perceived stress (PSS) scores, when compared to healthy controls, indicate the involvement of both psychological stress and immune mechanisms in the pathophysiology of CSU. The positive linear correlation between CSU severity and serum cortisol levels, as well as with impaired quality of life, further supports the association between stress and the clinical expression of CSU. Additionally, impaired quality of life positively correlated with perceived stress and negatively with age, highlighting the broader psychosocial impact of the disease, particularly in younger individuals.

All these indicate that psychological stress is associated with proinflammatory cytokines, which is important for CSU course and patient management. Taken together, these findings highlight that psychological stress significantly influences the course and severity of CSU and emphasize the need for a comprehensive, multidisciplinary approach towards the treatment of CSU patients. Since our study confirmed an association between proinflammatory cytokines IL-6 and TNF-alpha, psychological stress and CSU severity, this research may be a basis for further studies.

The manuscript is not under consideration elsewhere.

## Figures and Tables

**Table 1:** Comparison of variables between groups (median and interquartile range).

Parameter	Healthy Controls	Chronic Spontaneous Urticaria Patients	*p* *	r **
Age (years)	34 (27–48)	35 (29–48)	0.486	−0.115
IL-6 (pg/mL)	1.5 (1.5–2.7)	3.1 (1.9–4.7)	0.002	−0.460
TNF-α (pg/mL)	6.0 (4.3–7.7)	8.4 (6.4–10.3)	0.001	−0.485
Cortisol (nmol/L)	344.0 (273.0–364.0)	409.0 (322.0–476.0)	0.015	−0.360
Perceived stress (PSS, scalar points)	25 (21–29)	16 (12–22)	<0.001	−0.608

* Mann–Whitney test, ** effect size.

**Table 2:** Relationship between disease duration, disease severity, DLQI, serum IL-6, TNF-α, cortisol values, and age in CSU patients (assessed by Spearman correlation).

		Disease Duration	Disease Severity (UAS7)	Impairment of Quality of Life (DLQI)	IL-6	TNF-α	Stress (PSS)	Cortisol	Age
Disease duration (months)	r	1	0.092	−0.069	0.079	−0.081	−0.107	−0.115	0.174
	*p*		0.678	0.756	0.72	0.712	0.627	0.601	0.426
Disease severity (UAS7, scalar points)	r	0.092	1	**0.715**	0.214	−0.141	0.367	**0.463**	−0.323
	*p*	0.678		<0.001	0.328	0.520	0.085	0.026	0.132
Impairment of quality of life (DLQI, scalar points)	r	−0.069	**0.715**	1	0.113	0.018	**0.523**	**0.475**	**−0.529**
	*p*	0.756	<0.001		0.608	0.935	0.010	0.022	0.009
Serum IL-6 (pg/mL)	r	0.079	0.214	0.113	1	−0.235	−0.285	0.108	0.038
	*p*	0.72	0.328	0.608		0.28	0.187	0.623	0.863
Serum TNF-α (pg/mL)	r	−0.081	−0.141	0.018	−0.235	1	−0.030	−0.145	−0.229
	*p*	0.712	0.52	0.935	0.28		0.891	0.51	0.294
Perceived stress (PSS, scalar points)	r	−0.107	0.367	**0.523**	−0.285	−0.030	1	0.196	−0.133
	*p*	0.627	0.085	0.01	0.187	0.891		0.370	0.546
Serum cortisol (nmol/L)	r	−0.115	**0.463**	**0.475**	0.108	−0.145	0.196	1	−0.303
	*p*	0.601	0.026	0.022	0.623	0.510	0.370		0.159
Age (years)	r	0.174	−0.323	**−0.529**	0.038	−0.229	−0.133	−0.303	1
	*p*	0.426	0.132	0.009	0.863	0.294	0.546	0.159	

## Data Availability

The original contributions presented in this study are included in the article. Further inquiries can be directed to the corresponding author.

## Abbreviations

CU	chronic urticaria
QoL	quality of life
CSU	chronic spontaneous urticaria
TGF-β1	tumor growth factor-β1
MIP-1α	macrophage inflammatory protein 1α
RANTES	regulated upon activation, normal T-cell expressed and secreted
MCP-1	monocyte chemoattractant protein 1
DBC	differential blood count
ESR	erythrocyte sedimentation rate
CRP	C-reactive protein
IL	interleukin
TNF-α	tumor necrosis factor-alpha
SAM	sympatho-adrenal–medullary system
HPA	hypothalamic–pituitary–adrenal axis
CRH	corticotropin-releasing hormone
ACTH	adrenocorticotropic hormone (ACTH)
DLQI	Dermatology Life Quality Index
UAS	Urticaria Activity Score
ECLIA	electro-chemiluminescence immunoassay
PSS	perceived Stress Scale
